# The effect of episodic retrieval on inhibition in task switching: a diffusion model analysis

**DOI:** 10.1007/s00426-019-01206-1

**Published:** 2019-06-08

**Authors:** Agnieszka W. Kowalczyk, James A. Grange

**Affiliations:** School of Psychology, Dorothy Hodgkin Building, Keele University, Keele, ST5 5BG UK

**Keywords:** Task switching, Inhibition, Episodic retrieval, Diffusion model

## Abstract

Inhibition in task switching is inferred from $n-2$ task repetition costs: slower response times and poorer accuracy for ABA task switching sequences compared to CBA sequences, thought to reflect the persisting inhibition of task A across an ABA sequence. Much work has examined the locus of this inhibition effect, with evidence that inhibition targets response selection processes. Consistent with this, fits of the diffusion model to $n-2$ task repetition cost data have shown that the cost is reflected by lower estimates of drift rate, suggesting that inhibition impairs information processing efficiency during response selection. However, we have shown that the $n-2$ task repetition cost is confounded with episodic retrieval effects which masquerade as inhibitory costs. The purpose of the current study was to conduct a comprehensive analysis of diffusion model fits to new data within a paradigm that controls for episodic interference. Across four experiments (total $N = 191$), we find evidence that the reduction of drift rate for $n-2$ task repetition costs is only evident under conditions of episodic interference, and the cost is absent when this interference is controlled for. In addition, we also find evidence that episodic retrieval influences task preparation processes and response caution. These findings provide important constraints for theories of task switching that suggest inhibition selectively targets response selection processes.

Cognitive control refers to the set of cognitive processes that allow us to act in a goal-directed manner in the face of competing and/or ambiguous stimuli; it supports flexible and adaptive behaviour to changing task demands (Miyake et al., 2000), which is essential for goal-directed behaviour given our complex and busy environment. The task switching paradigm is an incredibly popular tool for investigating cognitive control processes (for reviews, see Grange & Houghton, 2014; Kiesel et al., 2010; Vandierendonck, Liefooghe, & Verbruggen, 2010). Within this paradigm, participants are required to make rapid responses to simple cognitive tasks on multivalent stimuli. For example, in the explicitly cued task switching paradigm (Meiran, 2014), participants might be presented with a digit stimulus and be asked to respond to whether the stimulus is odd/even or lower/higher than 5, with the currently relevant task being signalled by a task cue (e.g. the word “magnitude”).

One cognitive control process thought to assist task switching is the inhibition of recently performed tasks (Mayr & Keele, 2000). Evidence for such an inhibitory mechanism in task switching comes from paradigms in which participants are required to switch between three tasks (arbitrarily labelled A, B, and C). In such paradigms, it is a consistent finding that ABA task switching sequences are performed slower and with lower accuracy than CBA sequences. This so-called $n-2$*task repetition cost* is thought to reflect the persisting inhibition of task A across an ABA sequence: when switching initially from task A to task B, the persisting activation of task A interferes with the activation of the now-relevant task B; this interference is resolved by applying inhibition to task A which allows task B to become selected, and this inhibition persists for a short while which makes its reactivation on the final trial of the triplet less efficient (see Sexton & Cooper, 2017, for a computational model that formalises this account).

Considerable work has been conducted examining what influences the $n-2$ task repetition cost (see Gade & Koch 2014; Koch, Gade, Schuch, & Philipp, 2010, for reviews), with the aim of understanding what cognitive representations inhibition acts upon. For example, work has shown that inhibition can act upon cue-related processes (Gade & Koch 2014; Grange & Houghton, 2010; Houghton, Pritchard, & Grange, 2009; Scheil & Kleinsorge, 2014), stimulus-related processes (Sdoia & Ferlazzo, 2008), and response-related processes (Philipp, Jolicoeur, Falkenstein, & Koch, 2007; Schuch & Koch, 2003). This basic research programme has laid the foundations for utilisation of the $n-2$ task repetition cost to probe inhibitory control in clinical (e.g. Chen, Feng, Wang, Su, & Zhang, 2016; Fales, Vanek, & Knowlton, 2006; Foti et al., 2015; Mayr, Diedrichsen, Ivry, & Keele, 2006; Moritz, Hübner, & Kluwe, 2004; Whitmer & Banich, 2007) and healthy aging (e.g. Lawo & Koch, 2012; Mayr, 2001; Pettigrew & Martin, 2016; Rey-Mermet & Gade, 2018; Rey-Mermet, Gade, & Oberauer, 2017; Schuch, 2016) populations. As such, the $n-2$ task repetition cost has become valuable as a marker of cognitive inhibition in typical and atypical populations (Mayr, 2007).

The majority of work on inhibition in task switching has analysed $n-2$ task repetition costs utilising estimates of central tendency, such as mean response times and mean accuracy (but see Grange & Houghton, 2011; Schuch, 2016; Schuch & Konrad, 2017, for exceptions). However, two recent studies have fitted a version of the Ratcliff diffusion model (Ratcliff, 1978; Voss, Nagler, & Lerche, 2013) to $n-2$ task repetition cost data (Schuch, 2016; Schuch & Konrad, 2017). The diffusion model is an explicit computational account of the processes that lead to a response time in rapid decision-making tasks. As the diffusion model is a process model, it provides a richer account of the cognitive processes underlying response times than does analysing mean performance alone. As such, analysing $n-2$ task repetition cost data with the diffusion model arguably can lead to deeper insights into the nature of cognitive inhibition during task switching.

The purpose of the present article is to conduct a comprehensive analysis of diffusion model fits to $n-2$ task repetition data within a paradigm that controls for an important confound. Specifically, previous work from our lab has shown that much of the $n-2$ task repetition cost can be explained by memory interference during automatic episodic retrieval rather than inhibition. This work thus provides an important advance from the work of Schuch (2016) and Schuch and Konrad (2017) whose paradigm was not able to control for this confound. Our results are thus able to ascertain which of the findings from Schuch and colleagues replicate once episodic interference is controlled.

The remainder of the introduction is structured as follows. First, we provide an overview of the diffusion model. Then we provide an overview of the findings from Schuch and colleagues who applied the diffusion model to $n-2$ task repetition cost data. In the next section, we provide an overview of episodic retrieval and how it can explain much of the $n-2$ task repetition cost. We then present our experimental work.

## Diffusion modelling

**Fig. 1:**
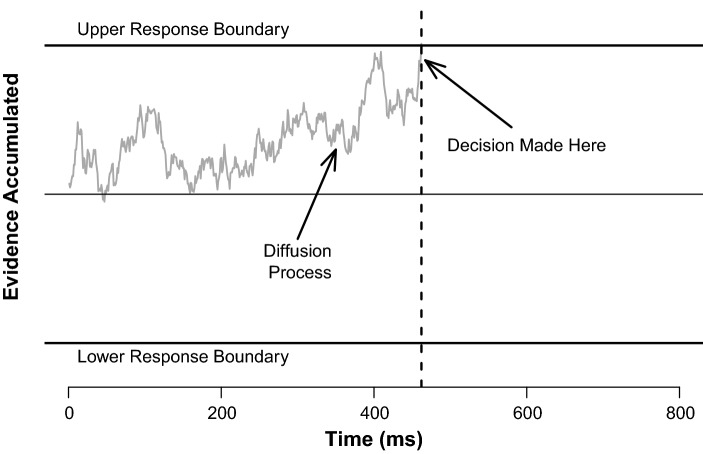
Schematic overview of trial processing in the diffusion model. Figure available at https://www.flickr.com/photos/150716232@N04/46893547582underCClicensehttps://creativecommons.org/licenses/by/2.0/

Figure 1 displays a schematic overview of the diffusion model. The model assumes that a response in a choice response time task is the result of a noisy evidence accumulation process (i.e. a diffusion process) whereby—once a stimulus is presented—evidence begins to accumulate in a noisy fashion towards one of two response boundaries; without loss of generality, one boundary represents the correct response, and the other boundary represents an incorrect response. This diffusion process continues until one of the two response boundaries is reached. The time taken for the diffusion process to reach this boundary reflects the decisional time of the model, and the boundary that is reached represents the model’s choice (i.e. whether the model’s choice was correct or incorrect). The diffusion model has three main parameters, which we discuss below.

### Drift rate

The drift rate represents the average rate of evidence accumulation during the diffusion process: higher values of drift rate reflect more efficient and rapid evidence accumulation toward a response. It is important to note that the drift rate reflects the average rate of evidence accumulation across all trials: stochasticity in the diffusion process leads to a different finishing time on each trial, which allows the diffusion model to account for response time distributions. Several internal (i.e. cognitive) and external factors can affect the magnitude of the drift rate, such as the perceptual quality of the stimulus itself (Voss, Voss, & Lerche, 2015), task difficulty, as well as individual differences such as working memory capacity and fluid intelligence (Schmiedek, Oberauer, Wilhelm, Suß, & Wittmann, 2007). In a traditional task switching context—that is, when assessing performance differences between immediate task repetitions and task switches between two tasks—it has been shown that estimates of drift rates are lower on task switch trials (Karayanidis et al., 2009; Schmitz & Voss, 2012), likely reflecting carryover effects from the previous (now irrelevant) task set. The drift rate is also sensitive to the degree of advance task preparation undertaken by the participant (Karayanidis et al., 2009; Schmitz & Voss, 2012). This is supported by the observation that drift rates are lower when participants have less time to prepare for the upcoming task (Schmitz & Voss, 2012, 2014) and that drift rates are higher when there is some degree of predictability to the task sequence (Karayanidis et al., 2009; Schmitz & Voss, 2012).

### Boundary separation

This parameter estimates the amount of evidence required before a response is executed; higher values of this parameter lead to slower (but more accurate) responses because the diffusion process takes longer to reach a higher boundary. This parameter is thought to be under the control of the participant, and can be set to meet speed–accuracy trade-off demands; it is thus a measure of the cautiousness of the participant. For example, in a task switching context, when the task design is predictable, participants are able to anticipate when the next trial will be relatively easy (e.g. when they expect an immediate task repetition), estimates of boundary separation have been shown to be lower (Karayanidis et al., 2009; Schmitz & Voss, 2012). Thus, when participants anticipate a task switch, they become more cautious with their responding.

### Non-decision time

This parameter captures the average time taken for stimulus encoding and motor responding on correct and error responses. However, recent task switching work has also suggested that the non-decision time parameter captures task preparation more generally, with faster non-decision time estimates reflecting more efficient task preparation, as estimates of non-decision time are longer under task switching conditions when task preparation was not possible (Schmitz & Voss, 2012, 2014).

## Diffusion modelling of $n-2$ task repetition costs

Only two studies to date have utilised diffusion modelling on $n-2$ task repetition cost data. Schuch (2016) investigated $n-2$ task repetition costs in healthy aging. There have been mixed results when examining age-related differences in the $n-2$ task repetition cost: whilst some studies have found larger $n-2$ task repetition costs in older adults (Mayr, 2001; Pettigrew & Martin, 2016), other groups have found no difference in costs (Lawo, Philipp, Schuch, & Koch, 2012; Rey-Mermet & Gade, 2018). Schuch (2016) addressed this by having a group of younger adults (18–26 years) and older adults (64–79 years) complete a task switching paradigm. Behavioural results showed no statistically significant difference in $n-2$ task repetition costs for response times or error rates. However, older adults showed the usual pattern of being slower but more accurate overall. The results of the diffusion modelling showed that $n-2$ task repetition costs in younger adults were reflected in the drift rate, with lower estimates for drift rate in ABA compared to CBA sequences, suggesting inhibition disrupts information processing during response selection. No other parameter showed an $n-2$ task repetition cost in younger adults. In older adults (when controlling for some outlier participants), the $n-2$ task repetition cost was reflected in all three main parameters: ABA sequences (compared to CBA sequences) led to lower drift rates, lower boundary separation, and longer non-decision times. The $n-2$ task repetition cost in drift rate was larger for older adults compared to younger adults.

Schuch and Konrad (2017) utilised diffusion modelling to examine $n-2$ task repetition costs in young children (9–11 years) compared to young adults (21–30 years). Similar to the study of Schuch (2016), there was no statistical difference in the $n-2$ task repetition cost in the behavioural data (either in response times or accuracy). For the drift rate, the results showed that adults had larger overall drift rates than children, suggesting more efficient response selection in adults. Importantly, adults showed a reduced drift rate in ABA compared to CBA sequences (replicating the finding of Schuch, 2016), which was not the case for children. For boundary separation, children had larger overall estimates than adults, suggesting a more cautious mode of responding in children. However, there was no clear difference between ABA and CBA sequences for either the adults or children on this parameter. For non-decision times, children had larger estimates than adults overall. Importantly (after controlling for outlier participants), children demonstrated longer non-decision time for ABA sequences compared to CBA sequences, which was not the case for adults.

## Interim summary

The results of Schuch (2016) and Schuch and Konrad (2017) are interesting for a number of reasons. One important aspect of their results worth emphasising is that in both studies there were no group differences in behavioural $n-2$ task repetition costs; group differences only emerged when analysing fits of the diffusion model. This demonstrates a key strength of diffusion modelling: group or experimental differences at the latent (i.e. model parameter) level might be masked if only analysing behavioural data.

Setting aside the specialist population results (i.e. children and older adults), the results for the younger adults in both studies are relatively consistent: $n-2$ task repetition costs appear in drift rates only. This suggests that task inhibition selectively disrupts information processing efficiency during response selection. This finding sits well with previous empirical work which has suggested that task inhibition leads to a lengthening of response selection processes (Schuch & Koch, 2003). For example, Schuch and Koch (2003) combined an $n-2$ task repetition paradigm with a go/no-go task which required participants to withhold a response on some trials. As the no-go signal was presented at the same time as the imperative trial stimulus, participants were not required to prepare or execute a response on these trials. The motivation for this manipulation was that if inhibition is triggered by response conflict between two task representations during response selection and/or response execution processes, then this conflict would not occur if the current trial was a no-go trial. In line with this view, Schuch and Koch (2003) found no $n-2$ task repetition cost if trial $n-1$ on an ABA sequence was a no-go trial, suggesting that task A did not become inhibited if the response for task B was not prepared or executed. From this point of view, the reduced drift rate in ABA sequences compared to CBA sequences found by Schuch (2016) and Schuch and Konrad (2017) reflects the carryover of the inhibition of response-related representations caused by inter-trial conflict, leading to slower and less efficient response selection.

## Episodic retrieval contributions to measures of inhibition

The $n-2$ task repetition cost has been well replicated across a wide range of different task designs (see, for example, Gade, Schuch, Druey, & Koch, 2014; Koch et al., 2010), and has been suggested to be a good measure of cognitive inhibition that is robust against non-inhibitory explanations (Mayr, 2007). However, recent work from our lab—extending the work of Mayr (2002)—has shown that a considerable portion of the $n-2$ task repetition cost is caused by interference during automatic episodic memory retrieval rather than inhibition. This account proposes that elements of a just-performed task (such as the perceptual characteristics of the cue, of the stimulus, the stimulus location, and also the motor response that was executed to this task) become bound together into a single memory representation (which Hommel refers to as an “event file”; Hommel, 1998, 2004). When this task is cued again (for example, in the final triplet of an ABA sequence), the most recent trace of this task is automatically retrieved from episodic memory (e.g. Logan, 1988, 2002). This automatic retrieval can lead to facilitation of performance if the elements of the retrieved trace match those of the currently presented trial (e.g. if the cue, the stimulus characteristics, and the required response match); however, this retrieval can lead to a relative cost to performance if there is a mismatch between the retrieved elements and those of the currently presented trial. This episodic mismatch can thus produce $n-2$ task repetition costs if trial parameters mismatch across an ABA sequence, leading to a mismatch cost.

**Fig. 2:**
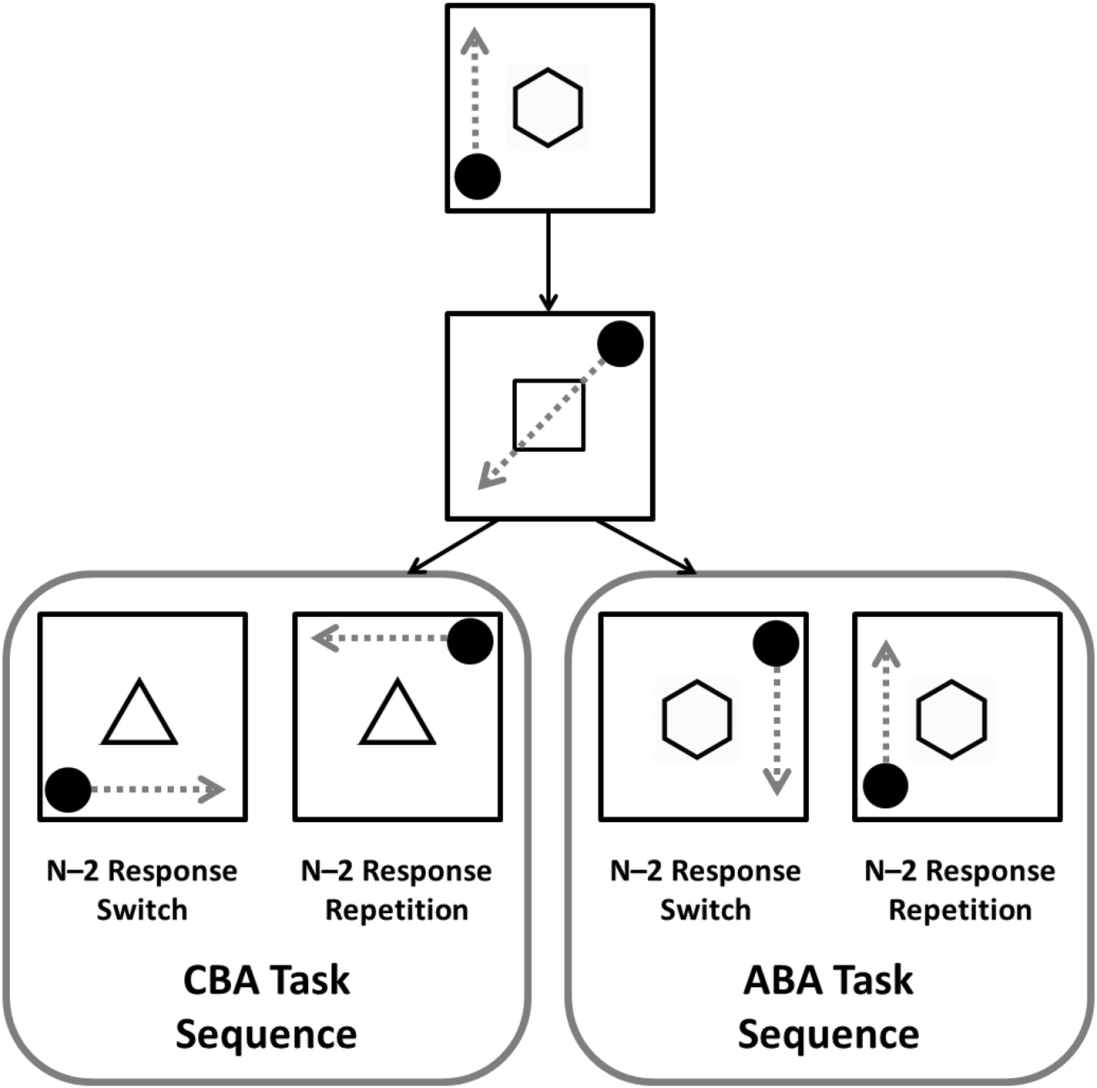
Schematic of the experimental paradigm. The arrows represent the spatial transformation required on each trial; these were not shown to participants. Time runs from the top to bottom of figure. Note that the image is not drawn to scale. Figure available at https://www.flickr.com/photos/150716232@N04/shares/5413G0underCClicencehttps://creativecommons.org/licenses/by/2.0/

To assess the contribution of episodic retrieval to the $n-2$ task repetition cost, Mayr (2002) designed the paradigm similar to that presented in Fig. 2. On each trial, participants need to mentally move a circular stimulus’ location according to one of three spatial rules (e.g. “horizontal”, “vertical”, or “diagonal”) indicated by a task cue (a hexagon, triangle, or square), and make a spatially congruent response as to where the stimulus would move to according to that rule. Within such a paradigm, binding on a trial would occur between the cue presented (e.g. “hexagon”), the stimulus location (e.g. “bottom-left”), and the response executed (e.g. “top-left”). In such a paradigm, we can control for which elements of this episodic trace repeat across an ABA sequence. For example, looking at the far-right of Fig. 2, on $n-2$ response repetitions, all elements of the trial match between trial n and trial $n-2$. Thus, on the current trial, the participant will retrieve from episodic memory a trace of the vertical task that matches the current trial elements (i.e. the cue is the same, the location of the stimulus is the same, and the response required is the same). This leads to facilitation of response selection. However, for $n-2$ response switches, there is a mismatch between the trial elements retrieved from episodic memory and the elements presented on the current trial (i.e. the cue is the same, but the stimulus is in a different location, and the required response is different), leading to longer response selection time. It is this lengthening of response selection time that can lead to $n-2$ task repetition costs from an episodic retrieval perspective. It is important to note that a pure inhibition account of the $n-2$ task repetition cost would predict similar $n-2$ task repetition costs for episodic matches and episodic mismatches because in both cases the relevant task (e.g. the vertical task) was inhibited at $n-2$.

Mayr (2002) found no statistical difference in the $n-2$ task repetition cost between episodic matches ($n-2$ response repetitions) and episodic mismatches ($n-2$ response switches). However, in the subsequent work (Grange, Kowalczyk, & O’Loughlin, 2017), we have found quite a substantial decrease in estimates of the $n-2$ task repetition cost for $n-2$ response repetitions (episodic matches) compared to $n-2$ response switches (episodic mismatches), a finding we have since replicated numerous times (Grange, 2018b; Grange, Kedra, & Walker, 2019; Kowalczyk, 2018). Despite this reduction in cost, we often find a small “residual” $n-2$ task repetition cost remains when controlling for episodic retrieval, suggesting some role for inhibition in task switching. Based on these results, we have suggested that the $n-2$ task repetition cost as typically measured is a confounded mixture of episodic interference effects and inhibition effects; any study wishing to make strong claims regarding inhibition needs to utilise a paradigm that can disentangle these two contributions to the cost.

## The current study

The purpose of the current study was to revisit the findings of Schuch (2016) and Schuch and Konrad (2017) of impaired drift rate for $n-2$ task repetitions in younger adults within a paradigm that controls for episodic retrieval effects. We wished to address whether the findings of reduced drift rate for $n-2$ task repetitions is caused by inhibition (as per Schuch and colleagues’ conclusions) or episodic interference, or a combination of both. We, therefore, fit the diffusion model to data from four experiments (three previously reported, and one new experiment) using the paradigm that controls for episodic retrieval effects.

## General method

All experiments shared a general method, which we describe below. We then describe the differences from this general method of all experiments. All experiments were approved by the Ethical Review Panel at Keele University.

### Participants

All participants were students at Keele University who either took part in exchange for partial course credit or for cash payment (£6). All participants were at least 18 years old, understood and spoke English, and had normal or corrected-to-normal vision. To be included in the final analysis, participants’ overall accuracy had to be at least 90%.

### Apparatus and stimuli

The task switching paradigm was presented on a standard PC with a 17 in. monitor via either E-Prime v.2.0 or PsychoPy v.2 (Pierce, 2007). Participants sat approximately 80 cm from the monitor. Responses were recorded via a 1-ms precise USB keyboard.

All experiments utilised a version of the task switching experiment depicted in Fig. 2. The stimulus frame consisted of a black 8-cm square grid presented on either a white or a grey background. The cues were either words (“horizontal”, “vertical”, “diagonal”) presented in black Verdana font, size 22 or black shapes with no fill (square, hexagon, triangle) approximately 3 cm in width and height. Responses were made on the numerical part of the keyboard using the 1, 2, 4, and 5 keys, which were spatially congruent with the response location (e.g. 1 = lower left; 2 = lower right; 4 = upper left; 5 = upper right). The stimulus was a filled circle measuring 1 cm in diameter.

### Procedure

The task required participants making rapid mental spatial transformations of the location of the circular stimulus according to one of three cued rules (“horizontal”, “vertical”, or “diagonal”), and to make a spatially congruent key press to the location that the stimulus would move to according to that rule. All data were collected in a single session within each experiment. The session lengths varied from 45 to 60 min, depending on experiment.

Prior to the testing session, participants learned the cue–task mappings, and took part in a brief practice block consisting of 16 trials. This practice block was repeated once if the participant made four or more errors. Each trial started with the presentation of the stimulus frame for 150 ms. After this time, the cue for the current trial appeared either in the centre of the frame, or above the frame (depending on experiment), for 150 ms, after which the stimulus appeared in one of the four inner corners of the frame. The cue and the stimulus remained on the screen until the participant made a response. The correct response was related to a spatial transformation of the stimulus’ position according to the currently relevant rule. For example, if the task was “horizontal” and the stimulus was presented in the bottom-left, the participant would move this stimulus in their mind to the bottom-right, and make a spatially congruent response (key = 2). After the response was registered, the cue and the stimulus were removed from the frame and the next trial began. If, however, the participant made a mistake, the word “Error!” would appear in red font in the cue’s location for 1000 ms. Participants were instructed to respond as quickly and as accurately as possible; they were instructed to use their index finger of their right hand to respond, and to reset their finger location to the mid-point of the four response keys after each response.

The cue for each trial was selected randomly with the constraint that no immediate task repetitions could occur. The stimulus location was chosen randomly on each trial.

#### Design

All experiments had the same design. Each experiment manipulated the within-subject factors of *Task Sequence* (ABA vs. CBA) and *Response* ($n-2$ response repetition vs. $n-2$ response switch). The dependent variables in all experiments were response time (RT) measured in seconds and proportion error.

### Individual experiment details

#### Mayr replication

This experiment—hereafter labelled the “Mayr” experiment—was a replication of Mayr’s (2002) experiment, and was reported in Grange et al. (2017; Experiment 1) and in Kowalczyk (2018). 76 participants met the inclusion criterion. This experiment was presented via E-Prime, and used word cues presented just above the stimulus frame (centred). After the practice block, participants were presented with four blocks of 120 trials with a self-paced rest screen after each block.

#### Working memory

This experiment—hereafter labelled the “WM” experiment—was part of an unpublished study (Kowalczyk, 2018) examining the relationship between the $n-2$ task repetition cost and working memory capacity. Participants took part in two sessions: in one session participants were presented with the task switching paradigm reported here, and in the other session participants took part in a battery of working memory capacity measures (which we do not report here). The order or task presentation was counterbalanced across participants. 42 participants met the inclusion criterion. The experiment was presented via E-Prime, and used shapes as cues. Participants were required to learn arbitrary cue–task pairings (e.g. “triangle” cue = horizontal task; counterbalanced) before the experiment began. The cues were presented centrally within the stimulus frame. After the practice block, participants were presented with four blocks of 120 trials with a self-paced rest screen after each block.

#### Healthy aging

This experiment—hereafter labelled the “aging” experiment—was a component of a study examining the effects of healthy aging on the $n-2$ task repetition cost (Grange et al., 2017). The data analysed here are from the younger adult control data. 29 participants met the inclusion criterion. The experiment was presented via PsychoPy using the same shape cues as the working memory study. After the practice block, participants were presented with eight blocks of 60 trials with a self-paced rest screen after each block.

#### New

Although the previous data sets have sufficient trial numbers for diffusion modelling (Voss et al., 2015), we wanted to bolster our findings by conducting a new study with increased trial numbers. To this end, we recruited 44 participants to take part in a new experiment—hereafter labelled the “new” experiment—presented via PsychoPy, using the shape cues from the WM and aging studies. After the practice block, participants were presented with 8 blocks of 120 trials with a self-paced rest screen after each block.

## Results

We wished to use an analytical strategy that combined the data from all four experiments. To this end, we utilised Bayesian multilevel modelling as our main analysis method, treating experiment as a random effect. Analysing the data from a multilevel perspective allows us to treat each experiment as a random sample from a population of experiments examining the effect of episodic retrieval on the $n-2$ task repetition cost; this allows us to make inferences beyond the sample of experiments presented here, something which standard fixed effects models (such as ANOVA) do not allow us to do. We supplement the multilevel modelling results with standard ANOVA for readers more familiar with this approach, but our inferences are based on the multilevel modelling.

The structure of results is as follows. First, we describe how we prepared our behavioural data for analysis and diffusion modelling. We then report the analysis of the behavioural data (i.e. RTs and error rates) to assess the effect of episodic retrieval on $n-2$ task repetition costs in the current data. We then describe the parameterisation and fitting procedure of the diffusion models, before formally assessing the goodness of fit of the models to our data. We then detail the inferential analysis of the diffusion model parameters.

### Data preparation

The statistical programming language R (R Core Team, 2017) was used together with various packages for data preparation, data analysis, and graphical visualisation. Specifically, we used the packages *dplyr* (Wickham & Francois, 2016), *tidyr*, (Wickham, 2018), *ggplot2* (Wickham, 2009), *trimr* (Grange, 2018a), *afex* (Singmann, Bolker, Westfall, & Aust, 2018), and *brms* (Bürkner, 2017).

The first two trials from each block were removed, as these cannot be classified as ABA or CBA sequences. For the response time analysis, error trials and the two trials following an error were removed. For the error analysis, just the two trials following an error were removed. Response times were trimmed by removing all RTs faster than 250 ms and slower than 5000 ms. Note that allowing RTs up to 5000 ms could be considered quite a liberal trimming approach; indeed, it is different (and more liberal) than how we trimmed our data in the original studies (e.g. Grange et al., 2017). However, it is important when modelling response time distributions that we do not trim too many data points from the tail of the distribution. In the next section, we show that this trimming procedure produces typical outcomes in the analysis. In Appendix 2, we explore whether a stricter response time trimming procedure alters the outcome of the analysis, and find that the results are qualitatively the same.

### Behavioural analysis

Before fitting the diffusion model, we assessed whether our modified trimming procedure altered the inferences previously made in the behavioural data, that is, we wished to assess the contribution of episodic retrieval to the $n-2$ task repetition cost in the response time and error data. To achieve this, we fit separate Bayesian multilevel models to the RT and error data, which we describe below. This analysis was supplemented by standard mixed-factorial ANOVA with the within-subject factors *Sequence* (ABA vs. CBA) and *Response* ($n-2$ response repetition vs. $n-2$ response switch), and the between-subject factor of *Experiment* (Aging vs. Mayr vs. New vs. WM); this ANOVA summary table can be seen in Table 1.

**Table 1:** Frequentist ANOVA summary tables for the behavioural effects in the dependent variables (DV) of response time and error

DV	Source	*df*	*F*	*p*	$\eta _G^2$
Response time	Sequence (S)	(1, 185)	133.55	< 0.001	0.02
	Response (R)	(1, 185)	44.91	< 0.001	< 0.01
	Experiment (E)	(3, 185)	6.30	< 0.001	0.09
	S $\times$ R	(1, 185)	83.31	< 0.001	0.01
	S $\times$ E	(3, 185)	0.80	0.50	< 0.01
	R $\times$ E	(3, 185)	1.82	0.15	< 0.01
	S $\times$ R $\times$ E	(3, 185)	2.42	0.07	< 0.01
Error	Sequence (S)	(1, 185)	14.38	< 0.001	0.01
	Response (R)	(1, 185)	7.71	< 0.01	< 0.01
	Experiment (E)	(3, 185)	9.97	< 0.001	0.09
	S $\times$ R	(1, 185)	61.84	< 0.001	0.03
	S $\times$ E	(3, 185)	1.62	0.19	< 0.01
	R $\times$ E	(3, 185)	0.58	0.63	< 0.01
	S $\times$ R $\times$ E	(3, 185)	4.52	< 0.01	< 0.01

#### Overview of Bayesian multilevel modelling

Bayesian multilevel modelling was conducted on both the response time and the error data, separately. In each analysis, four models were fit to the data, with each model differing in the inclusion of fixed-effect predictors of the dependent variable. Model 1 predicted the dependent variable from just a main effect of Sequence; Model 2 predicted the DV from just a main effect of Response; Model 3 predicted the DV from the inclusion of main effects of Sequence and Response; and Model 4 predicted the DV from the inclusion of two main effects plus their interaction. Default priors from the brms package were used for all models. All models had the same random effects structure, which had random intercept and slopes for the main effects of Sequence and Response for each participant nested within each experiment.^1^ For the RT analysis, a linear Gaussian model was used for the distribution of the dependent variable, whereas a beta response distribution (i.e. a beta regression) was used for the error analysis (because proportion error falls within the range [0, 1]).

Each model was fit to the data running four chains of the NUTS sampling procedure from the posterior distribution for each parameter. Each chain was set to sample 20,000 times from the posterior, with the first 10,000 samples being treated as burn-in. Visual inspection of the chains showed good convergence, and all $\hat{R}$ were close to 1.

Inference proceeded via model comparison to establish which model fits the data best. This was achieved by calculating the widely applicable information criterion (WAIC) for each model, which deals with the trade-off between model complexity and goodness of fit. Models with smaller WAIC values are to be preferred. We also calculated Akaike weights for each model, which provides an estimate of how likely each model is to provide superior predictions to new data sets in relation to other models in the set being compared. The Akaike weight for model *i* in relation to all models in the set *J* of models being compared is given by

1 $$\begin{aligned} \text {Weight}_{i} = \frac{\exp (-\,0.5 \times \text {dWAIC}_{i})}{\sum \nolimits _{j \in J} \exp (-\,0.5 \times \text {dWAIC}_{j})}, \end{aligned}$$

where dWAIC is the difference between model *i*’s WAIC and the best-fitting model’s WAIC. As this is a probability, models with weights closest to 1 are to be preferred. Once the best-fitting model was established, the posterior distributions of each population-level (i.e. fixed-effect) parameter were explored to make inferences.

#### Response times

**Fig. 3:**
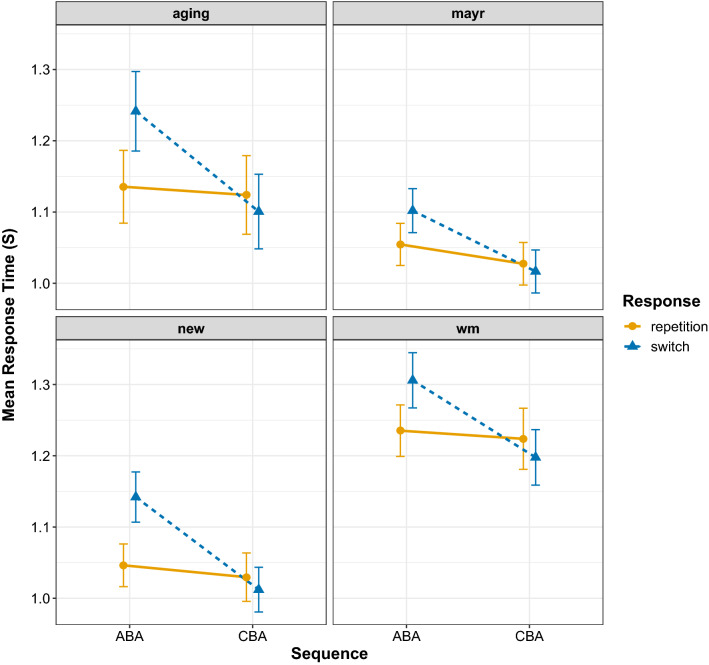
Mean response times (in seconds, s) for each data set. Error bars denote ± 1 standard error around the mean

Figure 3 shows the response time data for each experiment. As can be seen, in all experiments the $n-2$ task repetition cost was larger for $n-2$ response switches than for response repetitions (i.e. an interaction between Sequence and Response), replicating our earlier findings (Grange et al., 2017). Although the magnitude of the overall response speed differed by experiment, the main pattern in the data of an interaction between Sequence and Response was present in all experiments.

**Fig. 4:**
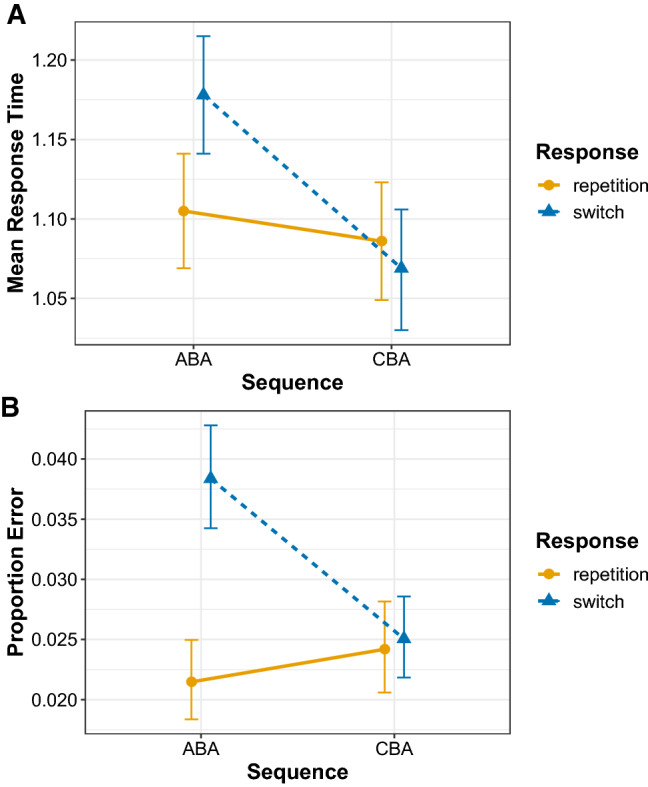
Population-level (i.e. fixed-effect) predictions from the best-fitting Bayesian multilevel model for response times (**a**) and proportion error (**b**). Error bars denote 95% Bayesian credible intervals around the mean estimates

The results of the model comparison procedure can be seen in Table 2. As can be seen, the best-fitting model was the model with two main effects plus their interaction with an Akaike weight of 1. The population-level parameters from this best-fitting model can be seen in Table 3, and the predictions from this model can be seen in Fig. 4a. As is clear from the figure and the model parameters, the $n-2$ task repetition cost is larger for $n-2$ response switches (110 ms, 95% Bayesian credible interval [99 ms, 120 ms]) than for $n-2$ response repetitions (19 ms [1 ms, 37 ms]), thus replicating our earlier findings (Grange et al., 2017).

**Table 2:** Model comparison results for the behavioural data. dWAIC shows the difference between each model’s WAIC and the best-fitting overall model’s WAIC, and Weight refers to Akaike’s weight for each model

Dependent variable	Model	WAIC	SE	dWAIC	Weight
Response time	Interaction (S $\times$ R)	− 1730	54	0	1
	Main effects (S + R)	− 1606	54	124	0
	Sequence (S)	− 1573	53	157	0
	Response (R)	− 1528	45	202	0
Error	Interaction (S $\times$ R)	− 4073	51	0	1
	Main effects (S + R)	− 4011	52	62	0
	Response (R)	− 4001	51	72	0
	Sequence (S)	− 3973	50	100	0

**Table 3:** Bayesian multilevel model parameters for the best-fitting model for each dependent variable for the behavioural data analysis

Dependent variable	Source	Estimate	Error	L-95% CI	U-95% CI
Response time	Intercept	1.11	0.02	1.07	1.14
	Sequence (CBA)	− 0.02	0.01	− 0.03	0.00
	Response repetition (switch)	0.07	0.01	0.06	0.09
	Interaction	− 0.09	0.01	− 0.11	− 0.07
Error rates	Intercept	− 3.82	0.08	− 3.98	− 3.67
	Sequence (CBA)	0.12	0.07	− 0.02	0.26
	Response repetition (switch)	0.60	0.07	0.46	0.74
	Interaction	− 0.56	0.09	− 0.74	− 0.39

Note that CI refers to lower (L) and upper (U) 95% Bayesian credible intervals

#### Error rates

**Fig. 5:**
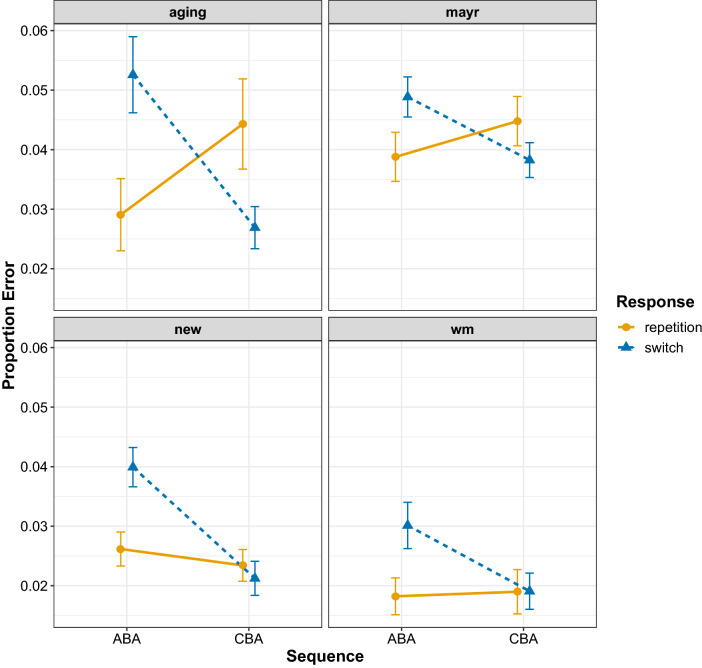
Mean proportion error for each data set. Error bars denote ± 1 standard error around the mean

Figure 5 shows the proportion accuracy data from each experiment; the ANOVA summary is in Table 1. The accuracy data also showed a consistent pattern of larger $n-2$ task repetition costs (i.e. poorer accuracy in ABA sequences compared to CBA sequences) for $n-2$ response switches than for response repetitions, that is, there was an interaction between Sequence and Response, which seemed to be further modulated by experiment. All experiments showed $n-2$ task repetition costs for response switches, but the pattern of the $n-2$ task repetition cost for response repetitions differed between experiments: the WM experiment showed no cost, the new experiment showed a slight cost, but the Aging and Mayr Experiments showed an $n-2$ task repetition *benefit*.

The results of the model comparison procedure can be seen in Table 2. As with the response times, the model with two main effects plus their interaction was the best model with an Akaike weight of 1. The population-level parameters from this best-fitting model can be seen in Table 3, and the predictions from this model can be seen in Fig. 4b. Again, the $n-2$ task repetition cost is larger for $n-2$ response switches (0.47 [0.36, 0.58]) than for $n-2$ response repetitions, where numerically there was an $n-2$ task repetition *benefit* (− 0.094 [0.067, − 0.255]).

### Diffusion modelling

The diffusion model was fit to the data using fast-dm-30 (Voss et al., 2015) which was called via custom scripts programmed in R.

#### Model parameterisation and fit procedure

The model was fit separately to each experiment’s data. In each experiment, we parameterised the model by allowing drift rate, boundary separation, and non-decision time to vary freely across the levels of the factors Sequence and Response. The starting point of the diffusion process was fixed halfway between the response boundaries (i.e. $z_{\mathrm{r}} = 0.5$). Following the advice of Voss et al. (2015), we fixed the trial–trial variability in drift rate ($s_{\mathrm{v}}$) and starting point ($s_{zr}$) to zero. Trial-to-trial variability in non-decision time ($s_{t0}$) was a free parameter, but was not free to vary across the levels of the factors Sequence and Response. Non-decision time was not allowed to differ for responses to the upper and lower thresholds (i.e. $d = 0$).

The model was fit to the data via maximum likelihood, which is recommended if trial numbers are relatively low (Voss et al., 2015). Table 4 shows the number of trials for each level of the design for each study; maximum likelihood is recommended if trials numbers are below 100, as is the case in three out of four of our experiments.

**Table 4:** Mean number of trials per participant per experiment after data trimming

Study	ABA repetition	ABA switch	CBA repetition	CBA switch
Aging	53	160	54	157
Mayr	55	167	55	168
WM	58	168	56	165
New	113	333	112	328

#### Goodness of fit assessment

Model fit was assessed via graphical inspection via QQ plots and Monte Carlo simulations—both of which are recommended by Voss et al. (2015).

*Graphical inspection* Graphical inspection of fit works by plotting observed data against simulated model predictions from the best-fitting parameters for each participant for each condition in each experiment. The data being plotted are the overall proportion accuracy, and the 25th, 50th, and 75th percentiles of the response time distributions from the participants and the simulated predictions. A good fitting model would produce simulated data across all moments (i.e. accuracy and response time distributions) that are close to the observed data; this leads to data points being clustered along the main diagonal in QQ plots. Appendix 1 provides more details on this method’s implementation, as well as the QQ plots.

*Monte Carlo simulation* The Monte Carlo approach establishes a criterion for which participants’ data were not fit well by the model. In the context of the fit routine reported here, a maximum likelihood value is obtained for each participant in each experiment, which represents the degree to which the model fits the observed data. Monte Carlo simulation allows us to establish a critical value for the maximum likelihood values, below which we can consider the model to have not fitted the participant’s data well.

The Appendix provides details of this method’s implementation. The outcome of this procedure was that the overall fit was excellent for all studies, as indicated by the percentage of participants who had a poor-fitting model: 0% in the Aging study; 1.35% in the Mayr study; 0% in the WM study; and 2.28% in the New study.

#### Inferential analysis

**Fig. 6:**
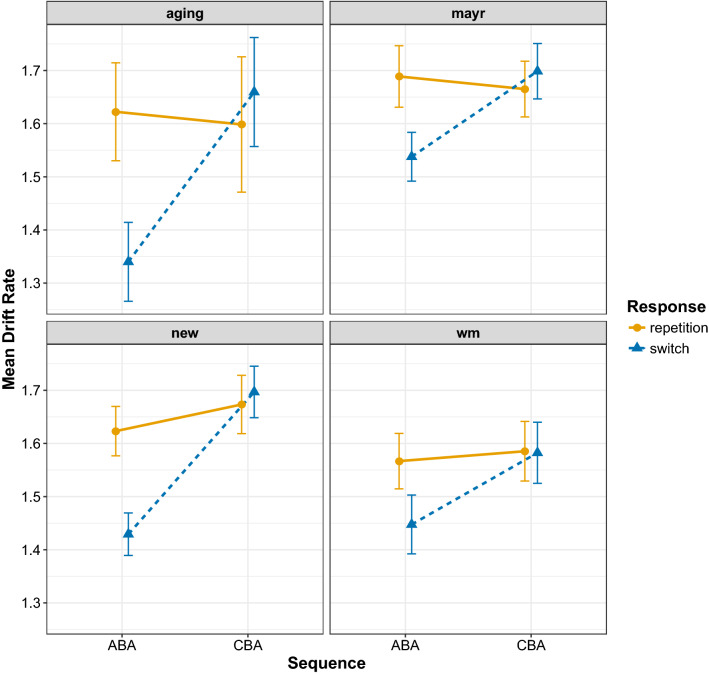
Mean estimates for the drift rate parameter for all data sets. Error bars denote ± 1 standard error around the mean

**Fig. 7:**
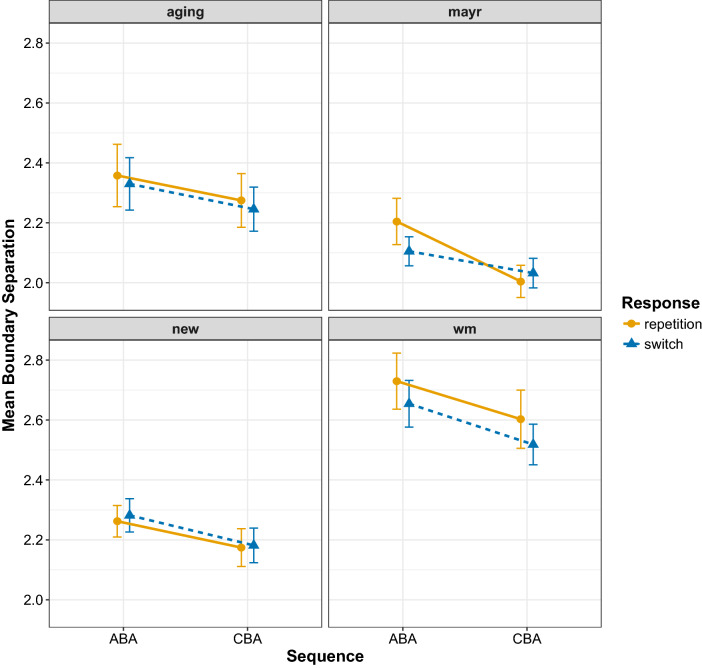
Mean estimates for the boundary separation parameter for all data sets. Error bars denote ± 1 standard error around the mean

**Fig. 8:**
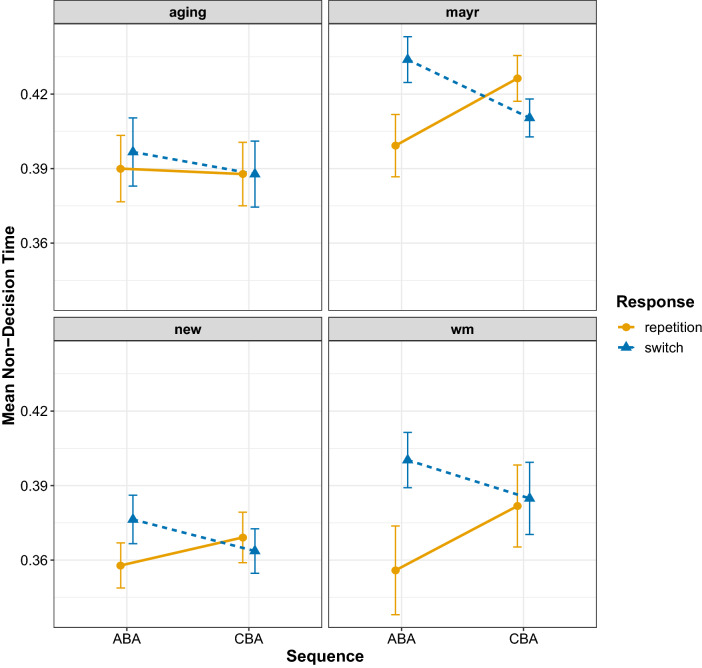
Mean estimates for the non-decision time parameter for all data sets. Error bars denote ± 1 standard error around the mean

The results of the diffusion model fits for the diffusion model parameters drift rate, boundary separation, and non-decision time are shown in Figs. 6, 7, and 8, respectively. To analyse these data, we again focus on Bayesian multilevel modelling. Each multilevel model had the same random effects structure as that reported for the behavioural data; all that changed was the dependent variable being entered into the analysis. For all models, a linear Gaussian model was used for the response distribution. Inference proceeded as for the behavioural data: for each diffusion model parameter separately, we constructed four multilevel models differing on the inclusion of fixed factors and their interaction; model selection again used WAIC and Akaike weight.

For the interested reader, the frequentist analysis of these data—consisting of a separate mixed-factorial ANOVA for each diffusion model parameter, with the within-subject factors *Sequence* and *Response Repetition*, and the between-subject factor *Experiment*—are shown in Table 5.

**Table 5:** Frequentist ANOVA summary tables for the diffusion model parameters

Parameter	Source	*df*	*F*	*p*	$\eta _G^2$
Drift	Sequence (S)	(1, 185)	39.39	< 0.001	0.02
	Response (R)	(1, 185)	27.96	< 0.001	0.01
	Experiment (E)	(3, 185)	0.82	0.49	0.01
	S $\times$ R	(1, 185)	50.68	< 0.001	0.01
	S $\times$ E	(3, 185)	1.90	0.13	< 0.01
	R $\times$ E	(3, 185)	0.61	0.61	< 0.01
	S $\times$ R $\times$ E	(3, 185)	1.98	0.12	< 0.01
Boundary	Sequence (S)	(1, 185)	25.70	< 0.001	0.01
	Response (R)	(1, 185)	2.21	0.14	< 0.01
	Experiment (E)	(3, 185)	14.93	< 0.001	0.15
	S $\times$ R	(1, 185)	0.42	0.52	< 0.01
	S $\times$ E	(3, 185)	0.37	0.77	< 0.01
	R $\times$ E	(3, 185)	0.77	0.51	< 0.01
	S $\times$ R $\times$ E	(3, 185)	1.00	0.39	< 0.01
Non-decision	Sequence (S)	(1, 185)	0.00	0.96	< 0.01
	Response (R)	(1, 185)	8.82	< 0.01	< 0.01
	Experiment (E)	(3, 185)	5.51	< 0.01	0.06
	S $\times$ R	(1, 185)	18.00	< 0.01	< 0.01
	S $\times$ E	(3, 185)	0.28	0.84	< 0.01
	R $\times$ E	(3, 185)	1.45	0.23	< 0.01
	S $\times$ R $\times$ E	(3, 185)	1.81	0.15	< 0.01

**Table 6:** Model comparison statistics for the Bayesian multilevel modelling of diffusion model parameters

Parameter	Model	WAIC	SE	dWAIC	Weight
Drift	Interaction (S $\times$ R)	− 75.24	52.29	0.00	1.00
	Main effects (S + R)	1.54	53.13	76.78	0.00
	Response (R)	22.29	48.41	97.53	0.00
	Sequence (S)	28.66	55.74	103.9	0.00
Boundary	Interaction (S $\times$ R)	369.50	103.58	0.00	0.43
	Main effects (S + R)	369.82	104.53	0.32	0.37
	Sequence (S)	370.99	106.32	1.49	0.20
	Response (R)	389.73	102.66	20.23	0.00
Non-decision	Interaction (S $\times$ R)	− 2262.32	65.57	0.00	1.00
	Response (R)	− 2225.62	66.17	36.70	0.00
	Main effects (S + R)	− 2224.20	66.00	38.12	0.00
	Sequence (S)	− 2213.69	67.55	48.63	0.00

**Table 7:** Bayesian multilevel model parameters for the best-fitting model for each dependent variable for the behavioural data analysis

Diffusion model parameter	Source	Estimate	Error	L-95% CI	U-95% CI
Drift rate	Intercept	1.64	0.03	1.58	1.69
	Sequence	0.00	0.02	− 0.04	0.05
	Response repetition	− 0.17	0.02	− 0.21	− 0.13
	Interaction	0.20	0.03	0.15	0.26
Boundary separation	Intercept	2.36	0.04	2.28	2.44
	Sequence	− 0.14	0.03	− 0.20	− 0.08
	Response repetition	− 0.06	0.03	− 0.11	0.00
	Interaction	0.04	0.04	− 0.03	0.12
Non-decision time	Intercept	0.38	0.01	0.37	0.39
	Sequence	0.02	0.01	0.01	0.03
	Response repetition	0.03	0.00	0.02	0.04
	Interaction	− 0.04	0.01	− 0.05	− 0.02

Note that CI refers to lower (L) and upper (U) 95% Bayesian credible intervals

**Fig. 9:**
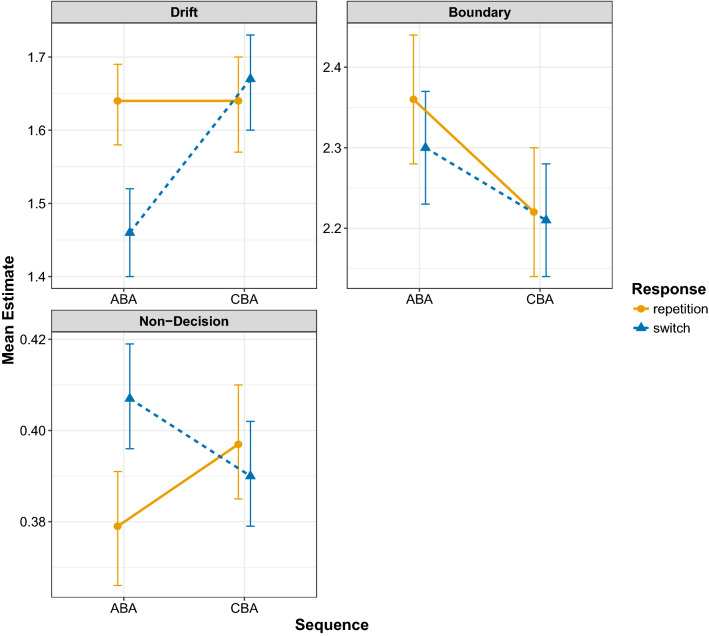
Population-level (i.e. fixed effect) predictions from the best-fitting Bayesian multilevel model for each diffusion model parameter. Error bars denote 95% Bayesian credible intervals around the mean estimates

Table 6 shows the outcome of the model comparison for all diffusion model parameters separately. For the drift rate parameter, the model with two main effects plus their interaction was preferred with an Akaike weight of 1. The population-level parameters of this model are shown in Table 7, and the predictions from this model are shown in Fig. 9. This analysis shows a clear $n-2$ task repetition cost—that is, lower estimates for drift rate for ABA sequences compared to CBA sequences—for $n-2$ response switches (− 0.204 [− 0.172, − 0.237]) that is altogether absent for $n-2$ response repetitions (− 0.006 [0.051, − 0.060]). This suggests that—in contrast to the findings of Schuch (2016) and Schuch and Konrad (2017)—the $n-2$ task repetition cost found in the drift rate is only apparent during episodic mismatches and is altogether absent when episodic matches occur.

For the boundary separation parameter, again the model with two main effects plus their interaction was preferred, but this time the Akaike weight (weight = 0.43) was low; the next-best-fitting model was the two main effects model (without the interaction) which had an Akaike weight of 0.37. This selection procedure suggests that whilst the interaction model should be preferred, its fit to the data is not convincingly superior to that of the two main effects model. Examining the population-level parameters of the interaction model (Table 7) and its predictions (Fig. 9) shows larger estimates for boundary separation for ABA compared to CBA sequences. This increase in boundary separation for ABA sequences compared to CBA sequences was slightly larger for $n-2$ response repetitions (0.139 [0.070, 0.210) than for $n-2$ response switches (0.095 [− 0.132, − 0.058]), but the evidence for this interaction is weak as indicated by the similar Akaike weight for the interaction and the main effects model, as well as the estimate for the interaction parameter in the Bayesian multilevel model ($\beta = 0.04$) which had a 95% Bayesian credible interval which included zero (− 0.03 to  0.12).

For the non-decision time parameter, the two main effects plus their interaction model was superior with an Akaike weight of 1. Examining the population-level parameters of the interaction model (Table 7) and its predictions (Fig. 9) shows an $n-2$ task repetition cost for $n-2$ response switches (0.017 [0.010, 0.024]), but an $n-2$ task repetition *benefit* for $n-2$ response repetitions (− 0.019 [− 0.031, − 0.007]); this analysis suggests that episodic mismatches produce an $n-2$ task repetition cost which turns into an $n-2$ task repetition benefit for episodic matches.

## General discussion

The purpose of the present study was to revisit the finding of Schuch (2016) and Schuch and Konrad (2017) who fit the diffusion model to $n-2$ task repetition cost data from task switching designs. They found that the $n-2$ task repetition cost was captured by a reduction of drift rate in ABA sequences compared to CBA sequences; this work supported the idea that inhibition selectively disrupts information processing during response selection stages of responding, which fits empirical findings from previous work from this group (see, e.g. Schuch & Koch, 2003; Koch et al., 2010). However, this work did not control for the effects of episodic retrieval, which we have shown can confound measures of the $n-2$ task repetition cost (Grange et al., 2017).

### Summary of results

In the current work, we fit the diffusion model to four data sets, all of which controlled for the effects of episodic interference. It is important to note that across these data sets we replicated the behavioural finding of reduced $n-2$ task repetition costs for episodic matches (i.e. $n-2$ response repetitions) than for episodic mismatches (i.e. $n-2$ response switches) for both response times and error rates. For the diffusion modelling—in contrast to the work of Schuch (2016) and Schuch and Konrad (2017)—we found $n-2$ task repetition effects in all three main parameters. We found that the $n-2$ task repetition cost was reflected by a reduction of drift rate in ABA sequences compared to CBA sequences, but only under conditions of episodic mismatch (i.e. $n-2$ response switches); there was no $n-2$ task repetition cost in the drift rate for episodic matches (i.e. $n-2$ response repetitions). This suggests that—in contrast to the conclusions of Schuch and colleagues—it is not inhibition that disrupts information processing during response selection, but rather it is episodic interference. We found that $n-2$ task repetitions increased estimates of the response boundary, which reflects response caution, that is, participants were more cautious in their responding for ABA sequences compared to CBA sequences. The model selection procedure suggested that this difference was slightly larger for episodic matches than for episodic mismatches, but the evidence for this interaction was not compelling. For the non-decision time parameter, clear differential effects of episodic retrieval were found on the $n-2$ task repetition cost: for episodic mismatches, there was an $n-2$ task repetition *cost* for this parameter, but this turned into an $n-2$ task repetition *benefit* for episodic matches. We elaborate on each parameter below.

#### Drift rate

That the $n-2$ task repetition cost for drift rate is modulated by episodic retrieval provides important constraints on theories of task inhibition in task switching. Schuch (2016) and Schuch and Konrad (2017) interpreted their finding of reduced drift rate for $n-2$ task repetitions compared to $n-2$ task switches as evidence for inhibition selectively impairing information processing during response selection processes. Our findings of strong episodic retrieval contributions to the drift rate suggest an alternative view, namely it is not the carryover of inhibition of response-related representations that reduces the efficacy of information processing during response selection, but rather it is the interference caused by the mismatch between the elements of the retrieved episodic trace and the elements of the currently presented trial.

#### Boundary separation

Although we found an $n-2$ task repetition cost for the boundary separation parameter, there was no clear modulation of this cost with episodic retrieval. This, therefore, suggests that participants were generally more cautious with their responding on ABA trials compared to CBA trials. This pattern was not found by Schuch (2016) and Schuch and Konrad (2017), but in our data it was robust, being present in all four data sets (Fig. 7). This parameter is thought to be under the control of the participant to balance speed and accuracy requirements (Voss et al., 2013). Although this finding suggests that $n-2$ task repetitions lead to more cautious responding, the reasons for this increase are not immediately clear. One possibility is that it is driven by a shift in response caution when the cognitive system registers interference on the current trial, either due to episodic interference or persisting inhibition (or both). However, as response caution is typically thought to be set by the participant *before* the trial (Voss et al., 2013), this account would suggest that the boundary separation can be changed dynamically in response to conflict, which remains speculative. Another—again, arguably speculative—account of increased response caution for $n-2$ task repetitions is that it reflects a violation of participant expectancy. For example, it has been proposed before that the $n-2$ task repetition cost could reflect a violation of participants’ expectations whereby participants expect CBA sequences to be more likely than ABA sequences; thus, when presented with an ABA sequence, the violation of expectancy leads to a slowing of responding (Koch et al., 2010; Mayr & Keele, 2000; Mayr, 2007). However, there is compelling evidence against this account of the $n-2$ task repetition cost; in particular, $n-2$ task repetition costs are observed even when participants are fully aware of the upcoming task sequence (e.g. Mayr, 2009). More work is thus required to explore what leads to an increase in response caution for $n-2$ task repetitions.

#### Non-decision time

Whilst Schuch (2016) and Schuch and Konrad (2017) found no $n-2$ task repetition cost for this parameter, we found an $n-2$ task repetition *cost* for episodic mismatches and an $n-2$ task repetition *benefit* for episodic matches. The opposing direction of the $n-2$ task repetition cost for episodic matches and mismatches (costs for mismatches; benefits for matches) might explain why Schuch (2016) and Schuch and Konrad (2017) found no $n-2$ task repetition cost for the non-decision parameter, as the cost and benefit would balance to a null effect if episodic retrieval is not controlled. The standard interpretation of the non-decision time parameter is that it reflects stimulus encoding and motoric response time (Voss et al., 2013); however, work in the task switching paradigm has also shown that this parameter also reflects general task preparation, with larger values reflecting less-prepared task preparation (Voss et al., 2015). Thus, an $n-2$ task repetition cost for episodic mismatches suggests that when the retrieved trace’s elements do not match the elements of the current trial participants are generally less-well prepared for responding. It could also be that the episodic mismatch leads to slower stimulus encoding time, which would also increase this parameter’s estimate; on episodic match trials, stimulus encoding should be facilitated because the current trial’s elements (which includes the stimulus and its position) match those retrieved from episodic memory, priming performance.

### Limitations

It is important to note some limitations of our modelling work and—potentially—the conclusions we draw from it.^2^ One potential concern is that the diffusion model assumes that the decision component (e.g. response selection) and the non-decision component (e.g. motoric responding) are discrete stages which do not occur in parallel. The concern raised by a reviewer was that this independence might be violated in our paradigm given its spatial-responding. Given the task requires the participant to move their finger to the spatially congruent response key, there is the possibility that the participant can lift their finger and move it (i.e. engage in motoric aspects) before completion of response selection. This would violate the assumptions of the diffusion model, and could explain why we find that the exclusive effect of $n-2$ task repetitions on drift rate found by Schuch (2016) and Schuch and Konrad (2017) spills over into other parameters in our study. Whilst this is certainly a possibility, we do not think that this issue is unique to our paradigm, so is unlikely to explain our results. For example, in typical task switching paradigms where just two response keys are used (as opposed to four in our study), it is typical in our experience to observe participants sometimes moving their fingers before a final response is executed. Thus, some degree of motoric action is very likely to occur in most choice response time tasks. Whilst this remains an interesting topic for diffusion model theorists to explore, we do not believe that the issue can uniquely explain our results of $n-2$ task repetition effects in other non-drift parameters.

Another issue raised by a reviewer was that it is perhaps not a correct assumption that the drift rate remains constant during trial processing in our paradigm. As the task requires the participant to mentally move the stimulus to another location and then to categorise this location, the system might treat this in a comparable way to a dynamically changing stimulus that produces an increasing drift rate the closer the (mental) stimulus gets to its shifted location. This would violate one of the assumptions of the basic diffusion model which has been fitted to our data.

We agree with the reviewer that this remains an interesting possibility. However, all cognitive modelling requires making simplifying assumptions which are (very likely) wrong in reality (Lewandowsky & Farrell, 2010), but these models can still be useful. In the present case, we fit a version of the diffusion model which assumes an unchanging drift rate during trial processing as this is the model which has been used in previous work on task switching (e.g. Schmitz & Voss, 2012; Schuch, 2016; Schuch & Konrad, 2017). We find quite different results to those found previously, and we acknowledge that this could be due to a violation of the simplified model’s assumptions of an unchanging drift rate. That our model fits the data incredibly well might speak against the possibility that the paradigm we have used violates a core assumption of the model. However, we should exercise some caution by not over-stating our results until this issue has been explored in more detail.

Future work should explore this modelling assumption in our current paradigm by developing and fitting diffusion models with an increasing drift rate. However, it is important to note that it is very likely that even a more complex version of the diffusion model would also be an over-simplification of the cognitive processes underpinning performance on our task. For one, it would not explain how task switching actually occurs. For this, one would need a richer process model, such as the computational model of Sexton and Cooper (2017). In previous work (e.g. Grange, 2018b), we have suggested that one promising line of research would be to explore how the formal computational model of inhibition in task switching by Sexton and Cooper (2017) could be extended to account for episodic retrieval effects, possibly by merging it with the Parallel Episodic Processing model of Schmidt, DeHouwer and Rothermund (Schmidt et al. (2016)). Modelling our episodic retrieval data in this manner can potentially lead to richer insights into the interplay of inhibition and episodic retrieval during task switching than any form of diffusion modelling can provide.

### Conclusion

These findings provide a challenge to theories of task switching that suggest inhibition selectively targets response selection and/or response execution processes (Koch et al., 2010; Schuch & Koch, 2003). Instead, our behavioural data support our previous conclusion that once episodic interference is controlled, there is a small (often absent) $n-2$ task repetition cost, suggesting a smaller role for inhibition than previously thought. The current work also provides some insight into the latent cognitive processes that give rise to the complex interaction between $n-2$ task repetition costs and episodic retrieval effects. For episodic mismatches, the diffusion modelling would suggest that the $n-2$ task repetition cost is driven by a combination of a reduction in task preparedness and/or less efficient stimulus encoding (as reflected by the non-decision time), a reduction in the efficiency of information processing during response selection, and an increase in response caution on ABA trials relative to CBA trials. For episodic matches, the diffusion modelling would suggest that we find a small, sometimes non-existent $n-2$ task repetition cost due to enhanced task preparation and/or more efficient stimulus encoding (as reflected by the non-decision time) which is somewhat offset by an increase in response caution for ABA trials relative to CBA trials.

## Figures and Tables

**Fig. 10:**
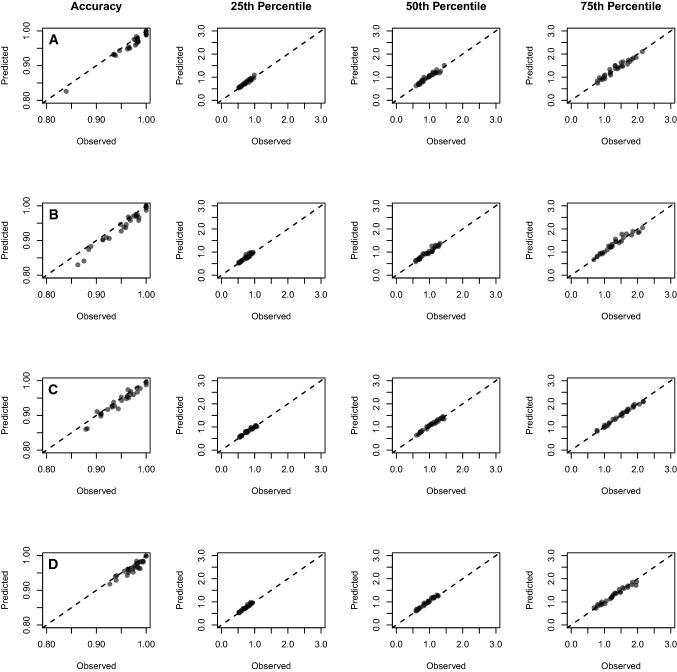
Graphical representation of the diffusion model fit to the “aging” data set. Each plot shows the observed data for each participant (each circle) plotted against the predicted data for the model for the accuracy (1st column) and for the 25th, 50th, and 75th percentiles of the response time distributions (columns 2–4) for four conditions (Rows A–D): Row A shows the ABA response repetition condition. Row B shows the CBA response repetition condition. Row C shows the ABA response switch condition. Row D shows the CBA response switch condition. The dotted diagonal line shows the line of perfect model fit

**Fig. 11:**
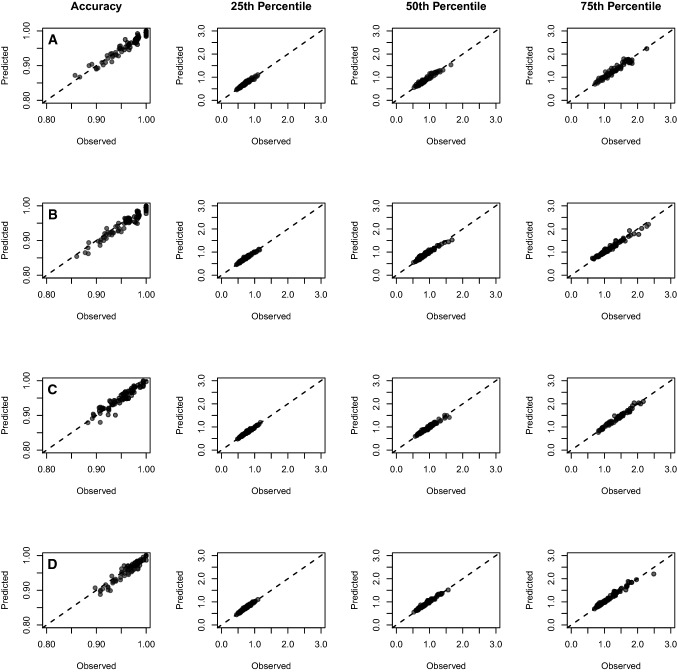
Graphical representation of the diffusion model fit to the “Mayr” data set. Each plot shows the observed data for each participant (each circle) plotted against the predicted data for the model for the accuracy (1st column) and for the 25th, 50th, and 75th percentiles of the response time distributions (columns 2–4) for four conditions (Rows A–D): Row A shows the ABA response repetition condition. Row B shows the CBA response repetition condition. Row C shows the ABA response switch condition. Row D shows the CBA response switch condition. The dotted diagonal line shows the line of perfect model fit

**Fig. 12:**
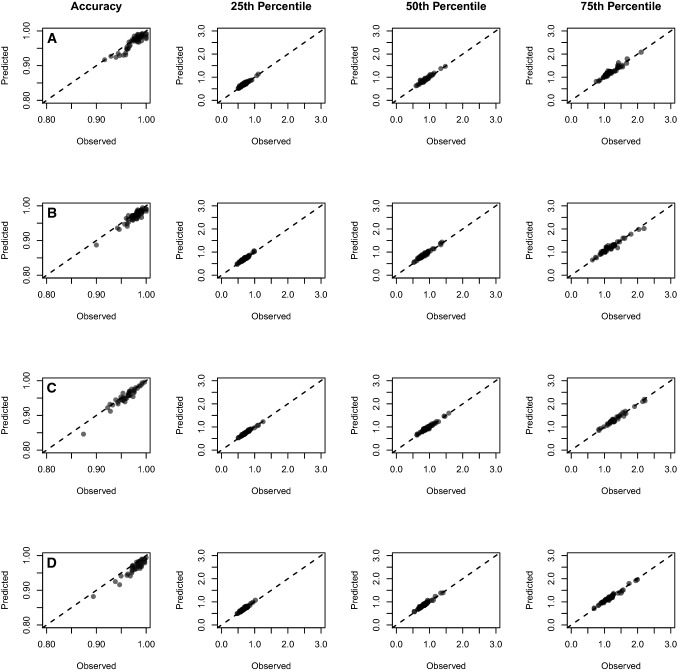
Graphical representation of the diffusion model fit to the “New” data set. Each plot shows the observed data for each participant (each circle) plotted against the predicted data for the model for the Accuracy (1st column) and for the 25th, 50th, and 75th percentile of the response time distributions (columns 2–4) for four conditions (Rows A–D): Row A shows the ABA response repetition condition. Row B shows the CBA response repetition condition. Row C shows the ABA response switch condition. Row D shows the CBA response switch condition. The dotted diagonal line shows the line of perfect model fit

**Fig. 13:**
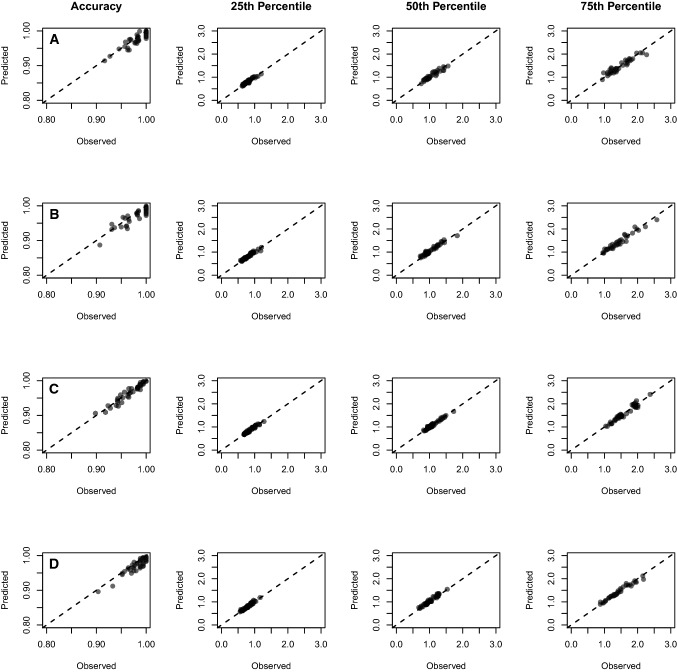
Graphical representation of the diffusion model fit to the “WM” data set. Each plot shows the observed data for each participant (each circle) plotted against the predicted data for the model for the Accuracy (1st column) and for the 25th, 50th, and 75th percentile of the response time distributions (columns 2–4) for four conditions (Rows A–D): Row A shows the ABA response repetition condition. Row B shows the CBA response repetition condition. Row C shows the ABA response switch condition. Row D shows the CBA response switch condition. The dotted diagonal line shows the line of perfect model fit

**Fig. 14:**
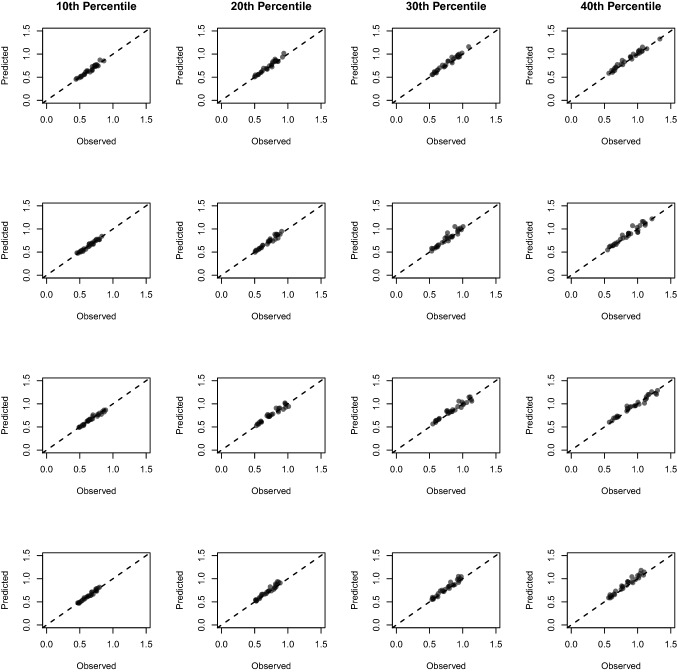
Graphical representation of the diffusion model fit to the “aging” data set. Each plot shows the observed data for each participant (each circle) plotted against the predicted data for the model for the 10th, 20th, 30th, and 40th percentiles of the response time distributions (columns 1–4) for four conditions (Rows A–D): Row A shows the ABA response repetition condition. Row B shows the CBA response repetition condition. Row C shows the ABA response switch condition. Row D shows the CBA response switch condition. The dotted diagonal line shows the line of perfect model fit

**Fig. 15:**
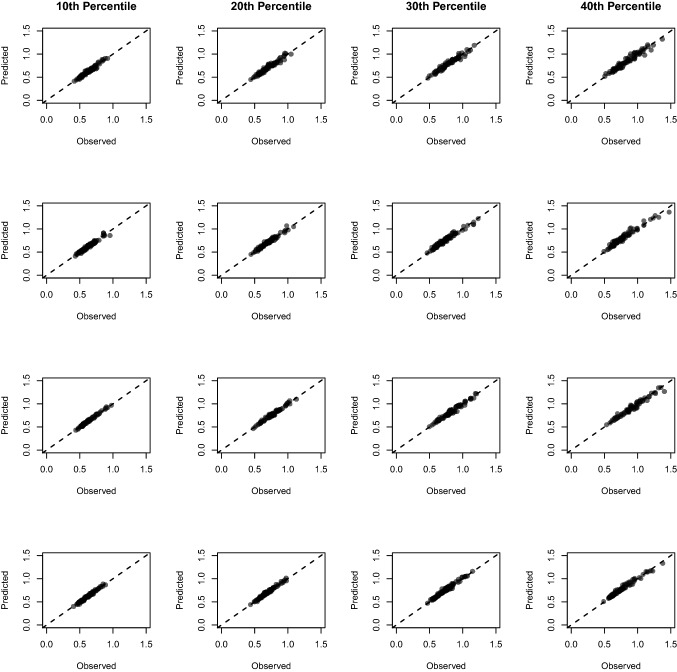
Graphical representation of the diffusion model fit to the “Mayr” data set. Each plot shows the observed data for each participant (each circle) plotted against the predicted data for the model for the 10th, 20th, 30th, and 40th percentiles of the response time distributions (columns 1–4) for four conditions (Rows A–D): Row A shows the ABA response repetition condition. Row B shows the CBA response repetition condition. Row C shows the ABA response switch condition. Row D shows the CBA response switch condition. The dotted diagonal line shows the line of perfect model fit

**Fig. 16:**
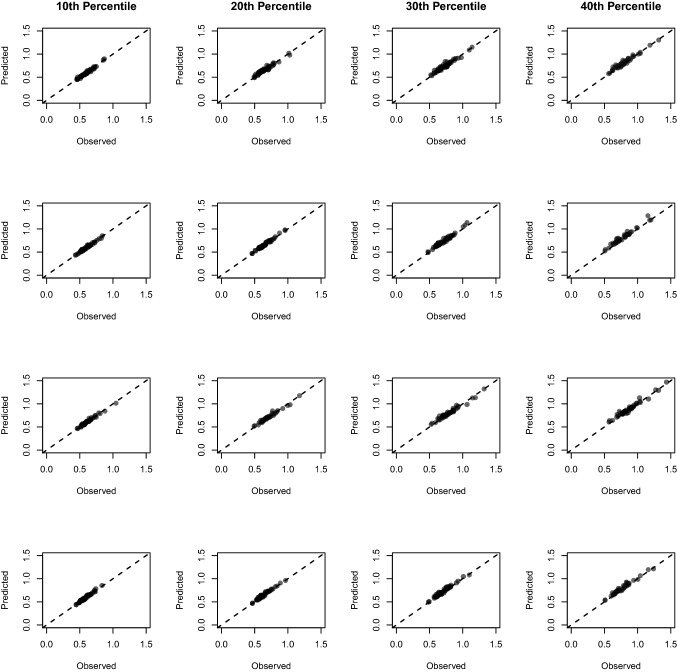
Graphical representation of the diffusion model fit to the “New” data set. Each plot shows the observed data for each participant (each circle) plotted against the predicted data for the model for the 10th, 20th, 30th, and 40th percentiles of the response time distributions (columns 1–4) for four conditions (Rows A–D): Row A shows the ABA response repetition condition. Row B shows the CBA response repetition condition. Row C shows the ABA response switch condition. Row D shows the CBA response switch condition. The dotted diagonal line shows the line of perfect model fit

**Fig. 17:**
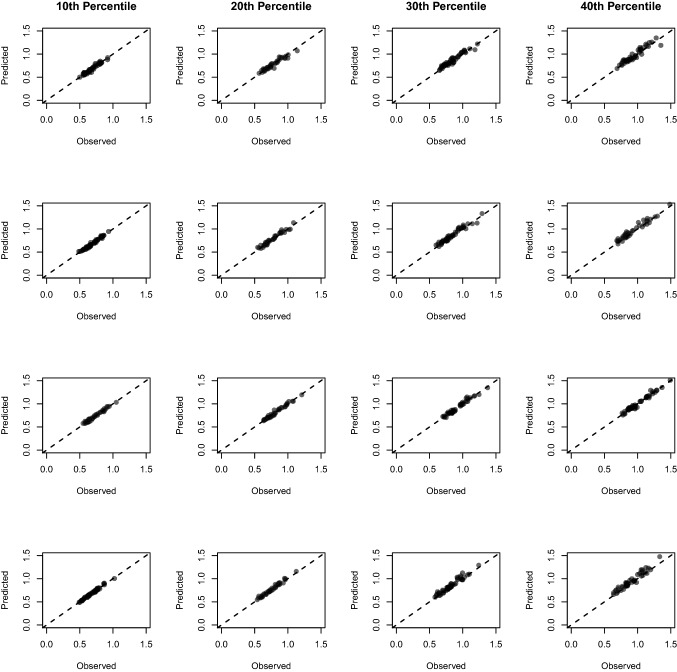
Graphical representation of the diffusion model fit to the “WM” data set. Each plot shows the observed data for each participant (each circle) plotted against the predicted data for the model for the 10th, 20th, 30th, and 40th percentiles of the response time distributions (columns 1–4) for four conditions (Rows A–D): Row A shows the ABA response repetition condition. Row B shows the CBA response repetition condition. Row C shows the ABA response switch condition. Row D shows the CBA response switch condition. The dotted diagonal line shows the line of perfect model fit

**Fig. 18:**
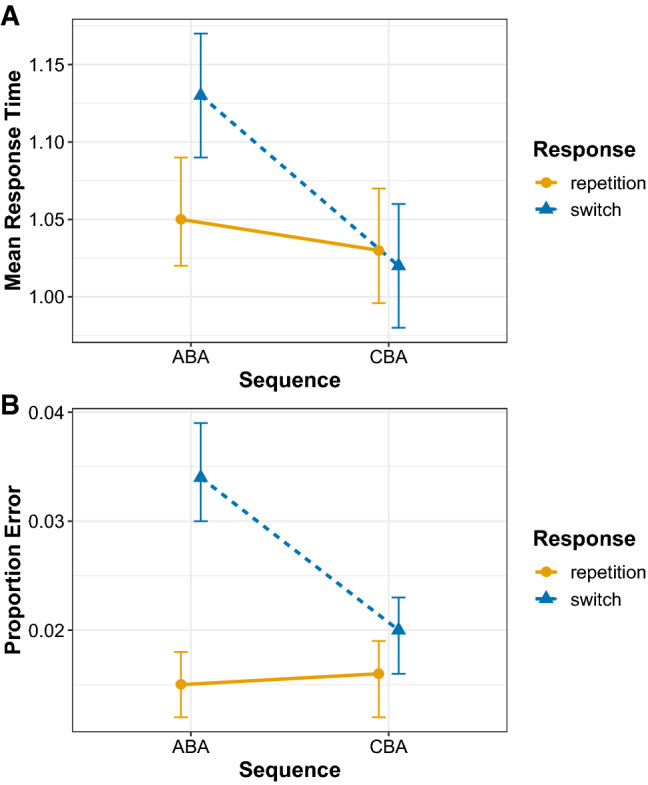
Population-level (i.e. fixed-effect) predictions from the best-fitting Bayesian multilevel model for response times (**a**) and proportion error (**b**) for the 2.5 SD trimming supplementary analysis. Error bars denote 95% Bayesian credible intervals around the mean estimates

**Fig. 19:**
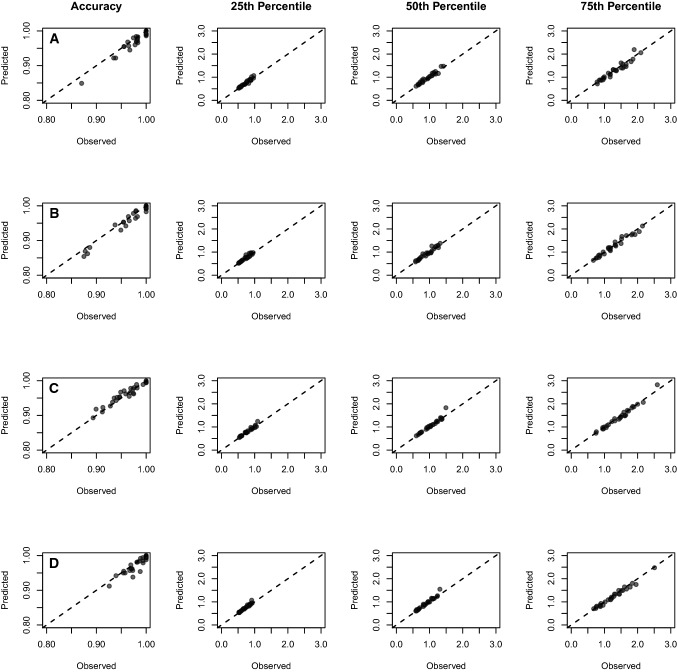
Graphical representation of the diffusion model fit to the “aging” data set. Each plot shows the observed data for each participant (each circle) plotted against the predicted data for the model for the accuracy (1st column) and for the 25th, 50th, and 75th percentiles of the response time distributions (columns 2–4) for four conditions (Rows A–D): Row A shows the ABA response repetition condition. Row B shows the CBA response repetition condition. Row C shows the ABA response switch condition. Row D shows the CBA response switch condition. The dotted diagonal line shows the line of perfect model fit

**Fig. 20:**
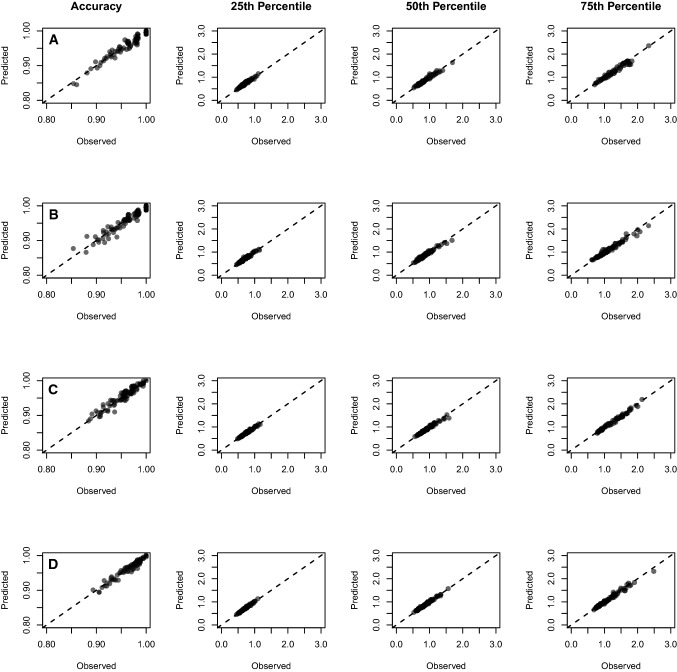
Graphical representation of the diffusion model fit to the “Mayr” data set. Each plot shows the observed data for each participant (each circle) plotted against the predicted data for the model for the accuracy (1st column) and for the 25th, 50th, and 75th percentiles of the response time distributions (columns 2–4) for four conditions (Rows A–D): Row A shows the ABA response repetition condition. Row B shows the CBA response repetition condition. Row C shows the ABA response switch condition. Row D shows the CBA response switch condition. The dotted diagonal line shows the line of perfect model fit

**Fig. 21:**
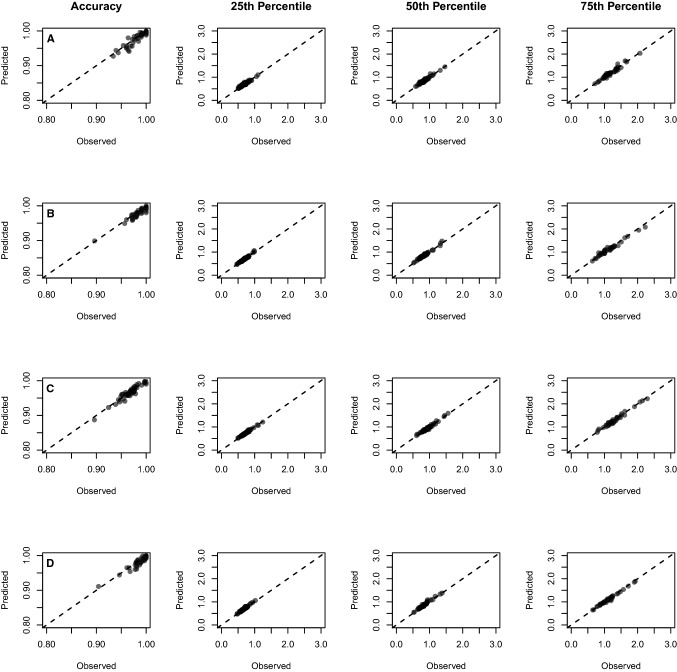
Graphical representation of the diffusion model fit to the “New” data set. Each plot shows the observed data for each participant (each circle) plotted against the predicted data for the model for the accuracy (1st column) and for the 25th, 50th, and 75th percentiles of the response time distributions (columns 2–4) for four conditions (Rows A–D): Row A shows the ABA response repetition condition. Row B shows the CBA response repetition condition. Row C shows the ABA response switch condition. Row D shows the CBA response switch condition. The dotted diagonal line shows the line of perfect model fit

**Fig. 22:**
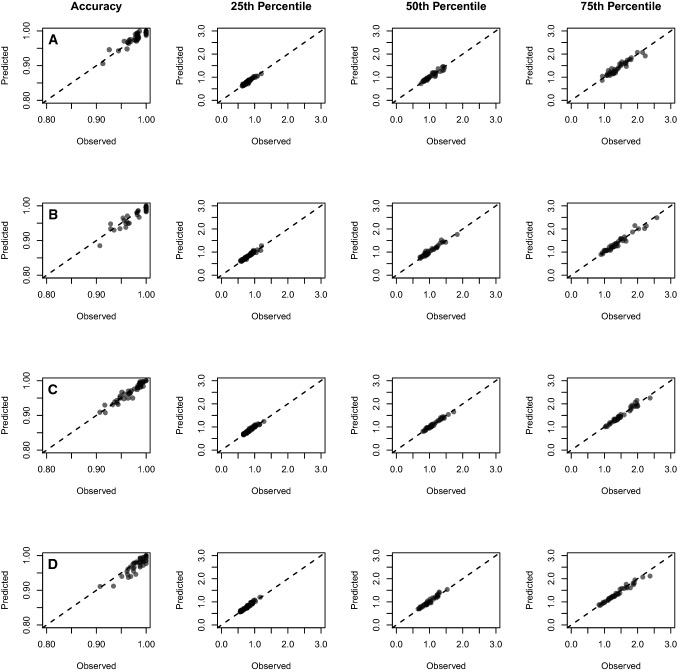
Graphical representation of the diffusion model fit to the “WM” data set. Each plot shows the observed data for each participant (each circle) plotted against the predicted data for the model for the Accuracy (1st column) and for the 25th, 50th, and 75th percentiles of the response time distributions (columns 2–4) for four conditions (Rows A–D): Row A shows the ABA response repetition condition. Row B shows the CBA response repetition condition. Row C shows the ABA response switch condition. Row D shows the CBA response switch condition. The dotted diagonal line shows the line of perfect model fit

**Fig. 23:**
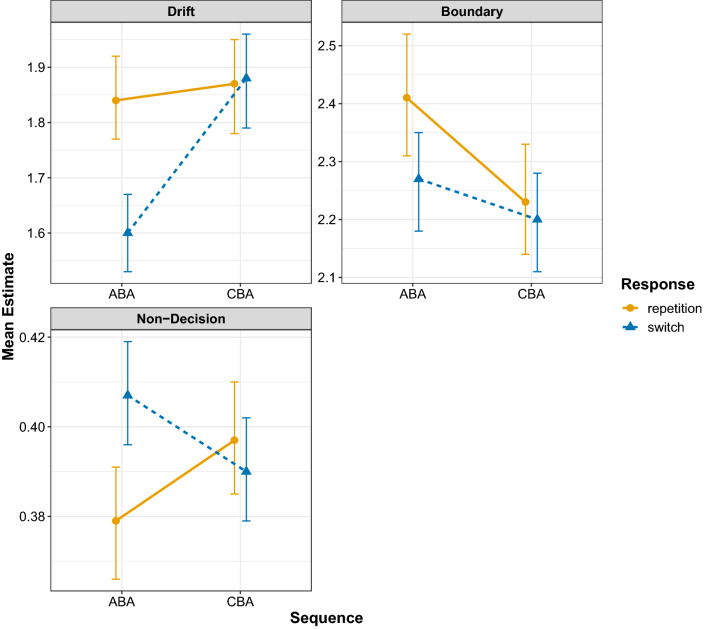
Population-level (i.e. fixed effect) predictions from the best-fitting Bayesian multilevel model for each diffusion model parameter for the standard deviation trimming analysis. Error bars denote 95% Bayesian credible intervals around the mean estimates

## Appendix 1: Assessment of model fit

### Graphical inspection of fit via QQ plots

Figures 10, 11, 12, and 13 show the graphical representations of model fits via QQ plots for all four experiments. The plots show model predictions against observed data for overall accuracy and for the 25th, 50th, and 75th percentiles of the response time distributions. Each row represents a different experimental condition (i.e. ABA repetition; CBA repetition; ABA switch; CBA switch).

The model’s predictions were obtained in the following way. Within a single experiment, each participant’s best-fitting parameters for each condition of the design (i.e. ABA repetition; CBA repetition; ABA switch; CBA switch) were extracted. Then, for each participant, 1,000 trials were simulated from the diffusion model separately for each level using the participant’s parameters (using the *construct samples* call in fast-dm-30). Then the proportion accuracy was calculated for the observed and the simulated data, as were the 25th, 50th, and 75th percentiles of the response time distribution, and these were plotted against each other. This was then repeated for each participant, for all conditions of the design.

All conditions showed a very good fit by the model, indicated by clustering of the data points along the diagonal line of perfect fit.

#### Lower RT quantiles

An anonymous reviewer of the paper suggested that examining the fit of the model at faster ends of the response time distribution allows for a better assessment of fit of the non-decision time parameter. To facilitate this suggestion, we provide QQ plots of the model fit at the 10th, 20th, 30th, and 40th percentiles for each condition for each data set in Figs 14, 15, 16, and 17. As can be seen, the fit of the model is very good.

### Monte Carlo analysis

For ease of exposition, below we describe the procedure for establishing the maximum likelihood criterion for one condition in one experiment only. The method is described in Voss et al. (2015), but was programmed independently in our analysis.

The method first takes all participants’ parameters for the condition, and then calculates the covariance matrix for the parameters. Then 1000 new parameter sets are generated by drawing from a multivariate normal distribution defined by the established covariance matrix. 1000 new data sets are then generated by taking each new parameter set and simulating data using these parameters from the diffusion model (again using the *construct samples* call in fast-dm-30). The number of trials to simulate is dictated by the average number of trials for that particular condition (see Table 4). Thus, each new data set is similar in size to our real participant data. Each of the 1000 simulated data sets are then fit with the diffusion model using our general fitting procedure reported in the main body of the paper. The maximum likelihood value of each fit is obtained and stored.

This procedure thus produces 1000 maximum likelihood values of diffusion model fits to data similar in structure (and with similar generating parameters) as our observed data. The 5% quantile of this distribution is then used as the criterion below which we consider a fit in our real data to be poor quality. The procedure then finds all participants in our fitting procedure who have maximum likelihood values lower than this criterion, and tags them as poor-fitting. Because the overall number of poor-fitting participants was very low (see main text), we did not remove any participants from the analysis.

## Appendix 2: Stricter response time outlier criterion

In this supplementary analysis, we checked the extent to which the primary findings were due to our choice of response time outlier criterion. Recall in the main analysis we excluded all RTs slower than 5 sec. We chose this value to ensure that the tail of the majority of participants’ response time distributions were retained to inform the modelling. However, it might be that our results depended upon this choice of outlier criterion, which is more lenient than typically found in the task switching literature.

We addressed this possibility by repeating our modelling after using a stricter RT trimming criterion. Response times were considered outliers if they were slower than 2.5 standard deviations above each participant’s mean RT for each cell of the experimental design. The criterion for fast outliers (250 ms) remained the same; we use this trimming criterion often (e.g. Grange et al., 2017). In this appendix, we first repeat our behavioural analysis to check the main findings remained the same before conducting the diffusion modelling.

### Behavioural analysis

The frequentist ANOVA for the new analysis can be found in Table 8. As in the main text, we focus on the Bayesian multilevel regression model comparisons, which can be found in Table 9.

**Table 8:** Frequentist ANOVA summary tables for the 2.5 SD trimming supplementary analysis of the behavioural effects in the dependent variables (DV) of response time and error

DV	Source	*df*	*F*	*p*	$\eta _G^2$
Response time	Sequence (S)	(1, 185)	120.77	< 0.001	0.02
	Response (R)	(1, 185)	46.89	< 0.001	< 0.01
	Experiment (E)	(3, 185)	6.35	< 0.001	0.09
	S $\times$ R	(1, 185)	71.61	< 0.001	< 0.01
	S $\times$ E	(3, 185)	0.97	0.41	< 0.01
	R $\times$ E	(3, 185)	2.05	0.11	< 0.01
	S $\times$ R $\times$ E	(3, 185)	2.23	0.09	< 0.01
Error	Sequence (S)	(1, 185)	16.25	< 0.001	0.01
	Response (R)	(1, 185)	7.30	< 0.01	< 0.01
	Experiment (E)	(3, 185)	13.88	< 0.001	0.12
	S $\times$ R	(1, 185)	49.62	< 0.001	0.03
	S $\times$ E	(3, 185)	1.41	0.24	< 0.01
	R $\times$ E	(3, 185)	0.54	0.66	< 0.01
	S $\times$ R $\times$ E	(3, 185)	1.68	0.17	< 0.01

**Table 9:** Model comparison results for the behavioural data for the 2.5 SD trimming alternative analysis

Dependent variable	Model	WAIC	SE	dWAIC	Weight
Response time	Interaction (S $\times$ R)	− 1623	66	0	1
	Main effects (S + R)	− 1521	64	102	0
	Sequence (S)	− 1485	62	138	0
	Response (R)	− 1448	56	175	0
Error	Interaction (S $\times$ R)	− 5084	126	0	1
	Main effects (S + R)	− 5042	127	42	0
	Response (R)	− 5032	126	52	0
	Sequence (S)	− 5008	125	76	0

dWAIC shows the difference between each model’s WAIC and the best-fitting overall model’s WAIC, and Weight refers to Akaike’s weight for each model

As in our main analysis, the best-fitting model for the response time analysis was the model with two main effects plus their interaction, with an Akaike weight of 1. The population-level predictions from this model can be found in Fig. 18a. As can be seen the $n-2$ task repetition cost is larger for $n-2$ response switches (0.108 [0.098, 0.119]) than for $n-2$ response repetitions (0.021 [0.001, 0.040]). This was true for the proportion error analysis, too (Fig. 18b). Here, the model with two main effects plus their interaction was also the best, with an Akaike weight of 1. The $n-2$ task repetition cost was 0.544 [0.414, 0.674] for $n-2$ response switches, being absent for $n-2$ response repetitions − 0.049 [− 0.215, 0.120] for $n-2$ response repetitions.

Thus, this stricter trimming method produced similar outcomes for the behavioural data.

### Diffusion modelling

The diffusion model was fit to this newly trimmed data in the same way as in the main text. The graphical inspection of the model fit via QQ plots is shown in Figs. 19, 20, 21, 22, which shows that the model fit the data particularly well.

We analysed the best-fitting model parameters in the same way as in the main text, via Bayesian multilevel regression model comparisons, the results of which can be found in Table 10. The population-level predictions of the best-fitting model for each diffusion model parameter can be found in Fig. 23. For the drift rate parameter, the model with the two main effects plus their interaction was the preferred model, with an Akaike weight of 1. As in the main analysis, the $n-2$ task repetition cost was large for $n-2$ response switches (− 0.272 [− 0.315, − 0.228]), and absent for $n-2$ response repetitions (− 0.025 [− 0.098, 0.049]). For the boundary separation parameter, the model with two main effects plus their interaction was the best model, with an Akaike weight of 0.76. The $n-2$ task repetition cost was larger for $n-2$ response repetitions (0.180 [0.070, 0.292]) than for $n-2$ response switches (0.070 [0.019, 0.121]). For the non-decision time parameter, the best model was again the one with two main effects plus their interaction with an Akaike weight of 1. The was an $n-2$ task repetition cost for $n-2$ response switches (0.019, 0.010, 0.027), but an $n-2$ task repetition *benefit* for $n-2$ response repetitions (− 0.022 [− 0.037, − 0.007]).

These findings confirm those of our main analysis.

**Table 10:** Model comparison statistics for the Bayesian multilevel modelling of diffusion model parameters for the 2.5 SD trimming alternative analysis

Parameter	Model	WAIC	SE	dWAIC	Weight
Drift	Interaction (S $\times$ R)	426.58	79.08	0.00	1.00
	Main effects (S + R)	483.92	79.06	57.34	0.00
	Sequence (S)	511.80	80.57	85.22	0.00
	Response (R)	512.55	72.81	85.97	0.00
Boundary	Interaction (S $\times$ R)	1001.12	119.09	0.00	0.76
	Main effects (S + R)	1003.49	119.73	2.37	0.23
	Response (R)	1012.81	118.34	11.69	0.00
	Sequence (S)	1013.70	122.30	12.58	0.00
Non-decision	Interaction (S $\times$ R)	− 1987.30	69.08	0.00	1.00
	Response (R)	− 1948.15	68.93	39.15	0.00
	Main effects (S + R)	− 1947.44	68.70	39.87	0.00
	Sequence (S)	− 1931.42	70.61	55.89	0.00

## Footnotes

1 In brms syntax, the random effect structure was (1 + Sequence + Response|Participant/Experiment).

2 We are grateful to an anonymous reviewer for raising some of these concerns.
